# Impaired Carbohydrate Metabolism among Women with Chronic Low Back Pain and the Role of Dietary Carbohydrates: A Randomized Controlled Cross-Over Experiment

**DOI:** 10.3390/jcm13072155

**Published:** 2024-04-08

**Authors:** Ömer Elma, Sevilay Tümkaya Yılmaz, Jo Nijs, Peter Clarys, Iris Coppieters, Evelien Mertens, Anneleen Malfliet, Tom Deliens

**Affiliations:** 1 Pain in Motion International Research Group, Department of Physiotherapy, Human Physiology and Anatomy, Faculty of Physical Education & Physiotherapy, Vrije Universiteit Brussel, 1090 Brussels, Belgium; sevilay.tumkaya.yilmaz@vub.be (S.T.Y.); jo.nijs@vub.be (J.N.); iris.coppieters@vub.be (I.C.); anneleen.malfliet@vub.be (A.M.); 2 Physiotherapy Unit, Department of Rehabilitation and Sport Sciences, Faculty of Health and Social Sciences, Bournemouth University, Bournemouth BH8 8GP, UK; 3 Chronic Pain Rehabilitation, Department of Physical Medicine and Physiotherapy, University Hospital, 1090 Brussel, Belgium; 4 Unit of Physiotherapy, Department of Health and Rehabilitation, Institute of Neuroscience and Physiology, Sahlgrenska Academy, University of Gothenburg, 40530 Gothenburg, Sweden; 5 Movement and Nutrition for Health and Performance (MOVE) Research Group, Department of Movement and Sport Sciences, Faculty of Physical Education & Physiotherapy, Vrije Universiteit Brussel, 1050 Brussels, Belgium; peter.clarys@vub.be (P.C.); evelien.mertens@ehb.be (E.M.); tom.deliens@vub.be (T.D.); 6 Research Foundation Flanders (FWO), 1000 Brussels, Belgium; 7 Nutrition and Dietetics Program, Department of Health Care, Design and Technology, Erasmushogeschool Brussel, 1090 Brussels, Belgium

**Keywords:** chronic pain, low back pain, pain sensitivity, postprandial glycemic response, carbohydrate intake, glycemic index, sucrose, isomaltulose

## Abstract

**Background:** Impaired glucose regulation is suggested to be related to chronic low back pain (CLBP), although it is not clear how they interact with each other. Thus, the primary aim of this study was to investigate differences in postprandial glycemic responses (PPGRs) (the first sign of impaired glucose metabolism) to high- (sucrose) and low-glycemic index (GI) (isomaltulose) beverages in normoglycemic women with CLBP and healthy controls (HCs) and explore whether any group that showed greater PPGRs to high-GI beverage intake would benefit when the high-GI beverage was replaced with a low-GI beverage. Secondly, this study aimed to explore the association between PPGR and pain in patients with CLBP. **Methods:** This study was registered at clinicaltrials.org (NCT04459104) before the start of the study. In this study, 53 CLBP patients and 53 HCs were recruited. After 11–12 h of fasting, each participant randomly received isomaltulose or sucrose. Blood glucose levels were measured during the fasting state and 15, 30, 45, 60, 90, and 120 min after the beverage intake, and each participant underwent experimental pain measures. **Results:** Compared to the HCs, the CLBP group showed significantly higher PPGRs to sucrose (*p* < 0.021). Additionally, the CLBP group showed a significantly higher decrease in PPGR (*p* = 0.045) when comparing PPGR to sucrose with PPGR to isomaltulose. Correlation analysis revealed a positive association between self-reported pain sensitivity and PPGR to sucrose, while there was no association found between any experimental pain measures and glycemic responses. **Conclusions:** Overall, these findings suggest that normoglycemic CLBP patients might have a higher risk of developing impaired glucose tolerance than the HCs and might benefit more when high-GI foods are replaced with low-GI ones.

## 1. Introduction

More than 15% of the population are diagnosed with low back pain (LBP) each year [1,2]. It is estimated that almost 10% of LBP conditions shift from an acute to a chronic phase and develop into chronic LBP (CLBP) [3]. The International Association for the Study of Pain (IASP) and the International Classification of Diseases (11th version) categorize pain as chronic when it persists for more than 3 months [4]. It has been determined that CLBP is the most prevalent type of chronic pain [5].

Similarly, type 2 diabetes mellitus (DM2) is considered a pandemic [6]. Impaired carbohydrate metabolism and DM2 are suggested to have an association with chronic pain and are suggested as risk factors for developing CLBP. Exposure to increased blood glucose levels may contribute to CLBP via various mechanisms, consisting of the direct impacts of glucose on the sensitivity of nociceptors [7], increased low-grade systemic inflammation subsequent to raised advanced glycation end products (AGEs), and oxidative stress [7], as well as atherosclerotic changes in the arteries of the spine leading to decreased blood supply [8]. Specifically, high-glucose conditions might have facilitating effects on central nervous system sensitization via dysregulating neuroinflammatory mechanisms [9]. To support this suggestion, research has demonstrated that metformin, a medication used to regulate blood sugar levels in diabetes mellitus, reduces inflammation and central nervous system sensitization (i.e., decreased sensitivity to mechanical and thermal allodynia) in addition to body weight [10]. Furthermore, individuals with impaired glucose tolerance and DM2 have a significantly higher incidence of chronic pain compared to those with normoglycemic status, further highlighting the link between glucose metabolism and pain perception [11].

One main factor that can affect carbohydrate homeostasis is diet. In a diet, the quality and quantity of ingested carbohydrates are the main factors dictating the effects of ingested foods on carbohydrate metabolism [12]. An elevated postprandial glycemic response (PPGR) is considered as the first sign of impaired carbohydrate metabolism [13]. The quantified amount of carbohydrates and the glycemic index (GI) value of carbohydrates in foods have been reported as being the best predictors of the PPGR [14]. In this sense, sucrose and isomaltulose are commonly used agents in exploring the reaction of carbohydrate metabolism to different GI carbohydrates, with cross-over trial designs being the most commonly employed methodology in this field [15,16]. Sucrose, as known as table sugar, has a high GI, whilst isomaltulose is classified as having a low GI due to its low digestion and absorption rate.

Overall, although the available literature suggests that DM2 might be associated with CLBP, it is not clear how CLBP and impaired carbohydrate metabolism interact with each other, and to date, there is no evidence demonstrating this interaction. Exploring the interaction between impaired PPGR as the first sign of impaired glucose metabolism and CLBP and intervening in this mechanism via nutritional strategies that target the quality and quantity of carbohydrates might have a strategic and promising impact not only on pain management but also on the management of dysregulated carbohydrate metabolism in this population. Thus, given the higher prevalence of CLBP in women and the recognized impact of sex on pain processing and glucose metabolism [3,17], our study exclusively included female participants and primarily investigated differences in PPGR to high- and low-GI beverages in women with CLBP and healthy pain-free controls (HCs) in a cross-over manner. Furthermore, we investigated the potential benefits of substituting high-GI beverages with low-GI beverages for any group exhibiting a higher PPGR to high-GI beverages. Lastly, this study delved into exploring the association between PPGR and pain-related outcome measures in patients with CLBP. We anticipate observing higher PPGRs in CLBP patients compared to the HCs. Additionally, we expect that individuals with CLBP will experience greater benefits from consuming low-GI beverages compared to high-GI beverages. Furthermore, we anticipate finding a positive correlation between PPGR and both experimental and self-reported pain measures in CLBP patients.

## 2. Materials and Methods

### 2.1. Study Design and Setting

This study was designed as a randomized controlled cross-over experiment and was conducted at Vrije Universiteit Brussel, Belgium, between September 2020 and December 2022. This trial was approved by the Medical Ethics Committee of the University Hospital (UZ Brussel; BUN1432020000025) on the 29 April 2020. The protocol of the study was registered at clinicaltrials.org prior to the start of the study (NCT04459104). This cross-over trial was reported according to the CONSORT (extension to cross-over trials) checklist [18].

**Figure 1:**
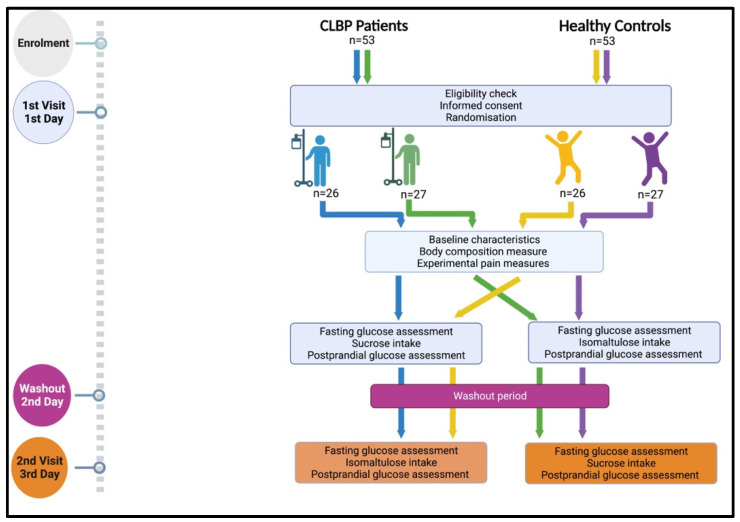
Flow of the study design (created with BioRender.com, accessed on 26 February 2024).

In this study, there were two groups, namely patients with CLBP and HCs. Each participant was individually randomized to receive both high- and low-GI beverages in a cross-over manner on two different days, with a one-day washout period in between. The flow of the study design is illustrated in Figure 1.

### 2.2. Sample Size Calculation

This study included 53 patients with CLBP and 53 HCs. The sample size calculation was performed based on the study of Tan et al. [15] using the same procedure to investigate the change in glycemic response. The sample size was performed in G*Power 3.1 (Düsseldorf, Germany) using the following inputs: two-tailed independent samples *t*-test, between-group differences, effect size (Cohen’s d) = 0.55, alpha set at 0.05, power of 0.8, and allocation ratio = 1.

### 2.3. Participants

Potential participants were reached via posters and flyers distributed in UZ Brussel and distributed to the general medical centers, pharmacies, and private physiotherapy clinics located around Brussels, as well as on the local social media channels (i.e., location-specific hashtags and geotags in Instagram and local community or interest-based Facebook groups). Eligible participants were invited to Vrije Universiteit Brussel, Health campus, Brussels, Belgium.

CLBP patients were included in this study if they met the following inclusion criteria: Dutch-speaking; aged between 18 and 65 years old; experiencing only non-specific CLBP for at least 3 months and at least 3 days per week; no analgesics/nicotine/caffeine/alcohol consumption 48 h prior to the assessments; no current pregnancy and no history of pregnancy in the last year; and not diagnosed with diabetes or any other systemic disease such as cardiovascular diseases. Additionally, people suffering from neuropathic pain, chronic widespread pain, or specific spinal pathology were excluded.

HCs were included in the study if they met the following inclusion criteria: Dutch-speaking; aged between 18 and 65 years old; no known health conditions; no analgesics/nicotine/caffeine/alcohol consumption 48 h prior to the assessments; no current pregnancy; and no history of pregnancy in the last year.

### 2.4. Procedure

This randomized controlled cross-over experiment consisted of two assessment sessions spread over two days with a one-day washout period in between (Figure 1). On the first day, participants with CLBP were screened for the presence of neuropathic pain using SLANSS and DN4 procedures (exclusion criteria) [19]. Then, participants went through body composition measurements followed by experimental pain measurements. Afterward, blood glycemic response analysis was performed, which included fasting blood glucose level assessment and blood PPGR measurement. This procedure was based on Tan et al. [15]. First, the fasting blood glucose level of the participants was measured. Then, participants were given a test beverage with a low or high glycemic index, after which the blood glucose level was measured at 15, 30, 45, 60, 90, and 120 min after the consumption of the test beverage.

After two days, participants were assessed for the second time, and only the fasting blood glucose level and PPGR were measured. Participants were given the other test beverage following fasting blood glucose level measurement. Then, the PPGR was measured at similar time intervals.

### 2.5. Randomisation and Blinding

Every participant in both groups was randomly given low- and high-GI beverages which were unidentifiable, as the beverages themselves looked similar and were given in similar-looking bottles. Both participants and assessors were blinded to the type of beverage given. Randomization of the participants, preparation of the beverages, and blinding were performed by an independent researcher who was not involved in the assessment, data collection, or statistical analysis.

### 2.6. Outcome Measures

#### 2.6.1. Baseline Characteristics

The following baseline characteristics were collected: age, use of medication, any existing health condition, duration of pain, date of diagnosis, used treatment modalities, physical activity, and quality of life levels.

#### 2.6.2. Blood Glycemic Response Measurements

Blood glucose levels were measured using OneTouch Verio (LifeScan Europe, Johnson & Johnson, Sug, Switzerland). OneTouch Verio uses a finger prick to collect a blood sample via test strips which are accurate and precise over a wide range of patients and environmental and pharmacologic conditions [20]. After a 10–12 h overnight fast period, two fasting blood drops were collected 5 min apart. If the difference between the two fasting blood glucose levels was more than 0.2 nmol/L, a third blood drop was collected. Then, participants were randomly given a test beverage with either a low (isomaltulose) or a high (sucrose) glycemic index. Then, 50 ± 0.01 g of sucrose (Kristalsuiker, Delhaize, Brussels, Belgium) or isomaltulose (Palatinose™, provided by BENEO, Brussels, Belgium) was measured on a calibrated electronic laboratory scale (AX124, Sartorius, Goettingen, Germany) and dissolved in 250 ± 0.1 mL of plain drinking water measured out with volumetric laboratory equipment. Afterward, blood glucose levels were collected at 15, 30, 45, 60, 90, and 120 min after consumption of the beverages. The first two drops of blood were discarded, and the third drop was used for testing. The same procedure was applied during the first and second assessment sessions.

#### 2.6.3. Experimental Pain Measures

##### Electrical Detection and Electrical Pain Thresholds

Electrical detection thresholds (EDTs) and electrical pain thresholds (EPTs) were used to estimate individuals’ sensitivity to electrical stimulation and assess their pain tolerance or threshold to such stimuli to evaluate pain perception and response. The Surpass LT stimulator (EMS Biomedical, Korneuburg, Austria) was utilized to measure EDTs and EPTs at four test locations, namely the bilateral median nerve and the bilateral sural nerve [21]. Between each of the experimental pain measures, there was a 5 min interval during which there was no stimulation to prevent contamination. The test site sequence was randomized to prevent sequencing bias.

For the median nerve test site, the cathode of the bipolar felt pad electrode was placed 5 cm proximally from the wrist, while the anodal electrode was placed 3 cm distally from the cathode. For the sural nerve test site, the surface electrodes for stimulation of the sural nerve were placed 2 cm posterior to the lateral malleolus. Each stimulus was a constant current rectangular pulse train consisting of 5 pulses delivered at a frequency of 250 Hz. Stimulation started at 0 mA and was gradually increased using steps of 0.5 mA until the patient experienced a faint sensation (=EDT) and further until the stimulus was experienced as painful (=EPT). Three measurements were made at intervals of thirty seconds, and the mean of the three measurements was utilized in all analyses.

##### Temporal Summation

In order to evaluate the endogenous pain facilitation, the temporal summation (TS) of electrical pain, a quantitative sensory test, was investigated [22,23]. Using the same randomization used to determine EDTs and EPTs, temporal summation was evaluated at the same four test locations. By applying 20 electrical stimuli at the EPT’s predetermined intensity, temporal summation was evaluated [22]. The patients were asked to give a verbal numeric rating scale (VNRS) score ranging from 0 (= no pain) to 100 (= worst possible pain) at the 1st, the 10th, and the 20th stimulus. The outcome measures for temporal summation were the differences between the 10th and 1st VNRS score, the 20th and 10th VNRS score, and the 20th and 1st VNRS score.

##### Electrical Offset Analgesia

Electrical offset analgesia (OA) is a type of quantitative sensory testing used to estimate the analgesic effect produced by a brief electrical stimulus which provides insights into the top-down pain inhibition mechanisms [24]. Electrical stimuli were applied as a train of rectangular pulses (frequency: 100 Hz; pulse duration: 1 ms) delivered by a constant current stimulator. The test site was located and marked 3 cm distally from the elbow joint on the volar side of the forearm of both the dominant and non-dominant arm. The stimulation intensity was calculated using the EPT. The study participants were given the painful stimuli in three times intervals and using three intensities: T1 (5 s at 150% of EPT), T2 (5 s at 180% of EPT), and T3 (20 s at 150% EPT). For safety reasons, stimulation did not reach above 50 mA. Afterward, participants underwent a control electrical stimulus which encompasses 30 s of constant electrical stimulation at 150% of the EPT. During each application (control trial, T1, T2, and T3), the patients were asked to give a VNRS score ranging from 0 to 100 every 5 s (at 4, 9, 14, 19, 24, and 29 s) after the onset of stimulation.

##### Pressure Pain Thresholds

The pressure pain threshold (PPT) is a measurement used to estimate the minimum pressure required to elicit pain in response to mechanical stimulation and measure hyperalgesia using a digital pressure algometer (Wagner Instruments, Greenwich) with a 1 cm^2^ tip [25]. The pressure was increased at a rate of 1 kg/s. Subjects were asked to say “stop” when the pressure was experienced as painful. PPTs were assessed at two different sites: a specific site for the CLBP group (i.e., bilaterally, 5 cm laterally to the L3 spinous process) and a distant reference point (i.e., tibialis anterior). A total of two measurements were collected from each area, separated by a 30 s interval, and averaged to reduce the measurement error. Then, the mean PPT value of the two measurements was calculated and used for the analysis.

#### 2.6.4. Anthropometry

Body composition was measured using a bioelectrical impedance analysis device (TANITA MC-780MA, Tanita Corp., Tokyo, Japan) and data was calculated and exported using GMON software version 3.4.2 (Medizin & Service GmbH, Chemnitz, Germany) [26]. The measured body composition components were body weight, body fat mass percentage, muscle mass percentage, water percentage, and body mass index (BMI). Body height was measured with a portable stadiometer (Seca^®^ 213, Hamburg, Germany).

#### 2.6.5. Self-Reported Questionnaires

##### Brief Pain Inventory (BPI)

The BPI enables respondents to score the worst pain level, the lowest pain level, the average pain level in the last 24 h, and the current pain level [27]. This instrument also uses an 11-point numerical rating pain scale, ranging from 0 (no pain) to 10 (worst pain), to measure how pain interferes with seven everyday activities: general activity, walking, work, mood, enjoyment of life, relationships with others, and sleep.

##### Central Sensitization Inventory

The two parts (A and B) of the Central Sensitization Inventory (CSI) are available for independent usage [28]. Only part A, which consists of 25 questions on a 5-point Likert scale from 0 (Never) to 4 (Always), was utilized in this section. A total score between 0 and 100 was obtained by adding the scores obtained from each of the 25 questions.

##### Physical Activity

Physical activity was measured by a validated Dutch version of the International Physical Activity Questionnaire (IPAQ) long form [29]. The IPAQ collects physical activity data under four main domains, namely “job-related physical activity”, “transportation”, “housework”, and “leisure”.

##### Quality of Life

Quality of life was measured using the Dutch version of the short form 36 (SF-36) quality of life questionnaire. SF-36 has 7 subscales, which are “physical functioning”, “social functioning”, “emotional health”, “bodily pain”, “mental health”, “vitality”, and “general health” [30].

### 2.7. Data Analysis

The statistical software SPSS (IBM Corp, Armonk, NY, USA) version 28.0 was used for the analyses. Minimum, maximum, mean values and standard deviation of the baseline clinical characteristics of the participants were calculated and reported with Cohen’s d effect sizes.

The normality of the data was evaluated using various graphical and formal statistical methods, including histograms, Q-Q plots, z-scores of kurtosis and skewness, and lastly the Kolmogorov–Smirnov test. One extreme outlier in the IAUC value of the PPGR to sucrose intake in the CLBP group was identified and Winsorized. The normality checks of the data revealed that the data were normally distributed. Thus, parametric statistical tests were used to investigate the primary and secondary hypotheses.

The positive incremental area under the curve (IAUC) of the PPGRs of each individual to sucrose and isomaltulose was calculated using the trapezoidal rule by ignoring the area under the fasting blood glucose level. This method involves dividing the area between the glucose curve and the baseline fasting blood glucose level into small trapezoidal segments and summing their areas. This calculation was performed by using the Python programming language (Python 3.8) and the source code with a table containing descriptions of the code elements can be found in Appendix A and Appendix B. After the identification of individual glycemic responses in IAUC, differences in postprandial sucrose and isomaltulose responses within and between the CLBP patients and pain-free HCs were analyzed using paired-sample *t*-tests and independent-sample *t*-tests, respectively. Additionally, the difference between the groups regarding the amount of change when replacing sucrose with isomaltulose was analyzed by applying an independent samples *t*-test after subtracting the IAUC isomaltulose value from the IAUC sucrose value. Lastly, associations between PPGRs (dependent variables) and pain outcome measures, body composition measures, physical activity levels, and diet quality scores (independent variables) were analyzed using Pearson’s correlation coefficient tests. *p*-values < 0.05 were considered statistically significant.

## 3. Results

Fifty-three females with CLBP and 53 HCs completed this study. There were no dropouts in this study. The baseline characteristics of the participants are shown in Table 1. Both the CLBP and HC groups did not differ statistically significant in age, anthropometry, mean fasting blood glucose levels, and certain experimental pain measures (EDT, EPT, TS, OA). Although the CLBP group had a higher score regarding their moderate activity level, both groups did not differ regarding their low, vigorous, and overall physical activity levels assessed by the IPAQ.

On the other hand, except for the social functioning and emotional health domains of the SF-36 quality of life questionnaire, the CLBP group showed significantly lower scores in the health-related quality of life assessed using SF-36. Moreover, the CLBP group had a significantly lower PPT value in the low back area (*p* < 0.001, Cohen’s d = −0.470) and a higher overall CSI score (*p* < 0.001, Cohen’s d = 1.110) compared to the pain-free HC group (see Table 1).

**Figure 2:**
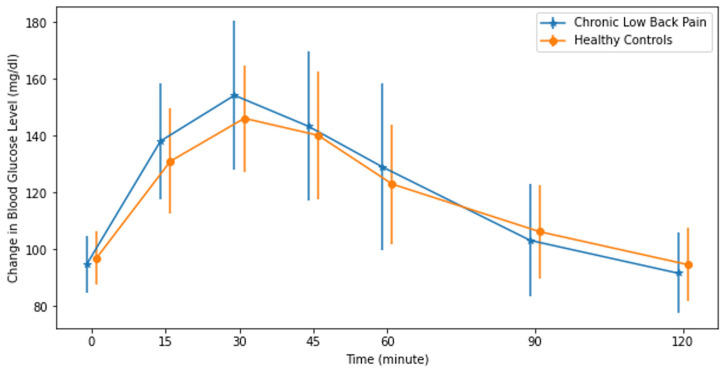
Mean and standard deviation of IAUC to 50 g of sucrose intake in the chronic low back pain and healthy pain-free control groups.

**Figure 3:**
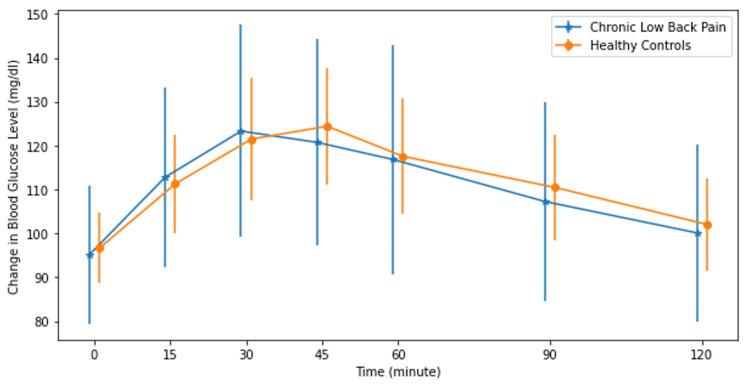
Mean and standard deviation of IAUC to 50 g of isomaltulose intake in the chronic low back pain and healthy pain-free control groups.

The PPGRs of the CLBP patients and HC group to oral sucrose and isomaltulose intake as expressed in IAUC are reported in Table 2. Within-group analyses using paired-sample *t*-tests revealed that both CLBP patients (*p* < 0.001, Cohen’s d = 0.959) and the HC group (*p* < 0.001, Cohen’s d = 0.628) showed statistically significantly higher PPGRs to sucrose intake compared to isomaltulose intake. After sucrose intake, the PPGRs of both CLBP and HC groups increased and peaked at the 30th minute and then gradually decreased (Figure 2). After isomaltulose intake, while the blood glucose level of CLBP peaked at the 30th minute, that of the HC group peaked at the 45th minute (Figure 3). None of the participants in either group had a 2 h PPGR value of more than 140 mg/dL, which is the minimum value for a prediabetic state. The PPGR to sucrose intake in IAUC was higher in the CLBP group (3470 ± 1525 min × mg/dL) than the HC group (2855 ± 1147 min × mg/dL) (*p* = 0.021, Cohen’s d = 0.959) (Table 3). The PPGR to isomaltulose did not show any significant difference (Table 3). When comparing PPGRs to sucrose and isomaltulose, both groups showed a statistically significant decrease in PPGR in IAUC, as the isomaltulose response was significantly lower than that to sucrose. However, the decrease in IAUC in the CLBP group (1380 ± 1375 min × mg/dL) was higher than in the HC group (844 ± 1344 min × mg/dL) (*p* = 0.045, Cohen’s d = 0.394) (Table 3).

In CLBP patients, the correlation analyses did not reveal any significant association between glycemic response measures (postprandial sucrose response, isomaltulose response, and amount of change when sucrose was replaced with isomaltulose) and any experimental pain outcome measures (electrical pain threshold, electrical detection threshold, temporal summation, offset analgesia, and pressure pain threshold). On the other hand, the PPGR to sucrose intake and the amount of change in PPGR were found to be positively associated with sub-components of BPI severity (average and least pain during the last 24 h) and interference (mood and sleep), but not with overall BPI severity and interference scores or the CSI as self-reported pain outcome measures. (Table 4).

## 4. Discussion

In this study, we primarily investigated differences in PPGRs to high- (sucrose) and low-GI (isomaltulose) beverage intakes in normoglycemic women with CLBP and pain-free HCs and explored whether any group that showed greater PPGRs to high-GI beverage intake would benefit when the high-GI beverage was replaced with a low-GI one. Secondly, we aimed to explore the association between PPGR and pain in patients with CLBP. Our findings primarily suggest that the CLBP group had considerably greater PPGRs to sucrose when compared to the HC group, but there was no difference between the two groups’ fasting blood glucose levels or PPGRs following isomaltulose ingestion. The CLBP group showed a more prominent decrease in PPGR when sucrose was replaced with isomaltulose. Furthermore, correlation analyses revealed a positive association between PPGR to sucrose intake and sub-components of BPI severity (average and least pain during the last 24 h) and interference (mood and sleep), but not with overall BPI severity and interference scores or any other pain measures. The findings of this study mainly highlight that the normoglycemic CLBP group might have a greater risk of developing impaired glucose tolerance compared to healthy controls due to elevated PPGR. Available evidence also demonstrates the increased risk of impaired glucose tolerance and even diabetes in the CLBP and in various chronic pain conditions, including chronic widespread pain [11,31,32]. For instance, in their observational cohort study including around 45,000 participants, Heucht et al. reported that women with CLBP showed a greater risk of developing DM2 with adjustments for age, BMI, physical activity, and smoking [31].

One potential factor affecting the elevated 2 h PPGR in the CLBP group could be insulin resistance, as in vivo studies on animal models and also human studies have shown a bidirectional association between chronic pain and insulin resistance [33,34]. Biomarkers of insulin resistance such as serum HbA1 level were suggested as remarkably positively associated with chronic pain and were even suggested as effective biomarkers in differentiating and classifying individuals with chronic pain among a control group [34]. Insulin resistance can exist among normoglycemic individuals with normal fasting glucose levels and is considered as a major cause of impaired glucose tolerance [35]. The presence of insulin resistance in the normoglycemic population was reported as being positively associated with 2 h PPGR over 100–139 mg/dL [36]. It was shown that 2 h PPGR, as assessed by IAUC, was substantially linked with elevated insulin resistance and risk of developing DM2, even in the range of 73 to 107 mg/dL [15]. This association is greater in individuals whose 2 h PPGR stayed above the fasting blood glucose level [37].

Besides PPGR, impaired carbohydrate metabolism can also be identified with fasting blood glucose level measurement. As only normoglycemic participants were included in the present study, it was not surprising that it was not possible to identify any statistically meaningful differences in the fasting blood glucose levels of both groups. Prior to an impaired fasting blood glucose level, elevated PPGR is considered a first sign of impaired carbohydrate metabolism in normoglycemic individuals [13]. Moreover, compared to the fasting blood glucose level, the PPGR has been reported as a better predictive factor in the risk of developing cardiovascular diseases in the diabetic population [38]. Thus, it is possible that only an elevated PPGR was identified as the first sign of impaired carbohydrate metabolism in the normoglycemic CLBP group.

In our study, we also found that consuming low-GI carbohydrates, namely isomaltulose, had little to no effect on the overall PPGR in individuals with CLBP. This low-GI carbohydrate intake effectively eliminated the difference in PPGR between the CLBP group and the HCs when compared to high GI carbohydrate ingestion. There are two main methods used to measure postprandial glycemic response, namely measuring the oral glucose load (i.e., as used here) and assessing mixed meals also containing fat and protein. It has been shown that both methods reveal similar findings in circulating glucose, insulin, and glucose uptake rates [39]. In this sense, information gathered from the oral glucose load and mixed meals reveal similar findings regarding the postprandial glucose mechanism [39]. Thus, the consumption of low-GI foods in general may have no/little effect on the overall PPGR among CLBP and eliminate the difference between the CLBP group and healthy controls.

Regarding pain-related outcome measures, we only identified a positive association between glycemic response (PPGR to sucrose and amount of change in PPGR when sucrose is replaced with isomaltulose) and components of BPI severity (average and least pain during the last 24 h) and interference (mood and sleep). We could not identify any association between any other pain outcome measures (i.e., experimental pain measures, CSI, and overall BPI severity and interference scores) and carbohydrate metabolism. Firstly, it is possible that the study was underpowered for the secondary research question. In fact, a posteriori sample size calculations d showed that the sample sizes needed for the correlation analysis between pain outcome measures and glucose metabolism were bigger than the actual recruited number of participants. However, due to small to very small effect sizes (Pearson r-coefficients), we do not expect this to have influenced our conclusions. Secondly, this may also be partially explained by the low levels of interference (3.1 ± 2.4) and pain severity (3.3 ± 1.9) in the CLBP patients, who also did not differ from pain-free HCs in experimental pain measures, including EDT, EPT, TS, OA scores, and PPT of the tibialis anterior. Although the CLBP group had significantly higher CSI scores, almost half of the patients (*n* = 24) had CSI scores below 40, and the mean CSI score was only 42. Remarkably, CLBP patients displayed local hyperalgesia via a decreased PPT in the low back area compared to pain-free HCs. On the other hand, we identified a positive association between self-reported pain sensitivity and glycemic response in CLBP patients, although the current literature also lacks evidence to support a causal relationship between the two conditions. In line with our findings, it is revealed that even in healthy individuals, acute hyperglycemia can interfere with pain processing mechanisms and result in an increase in pain sensitivity [40]. There are potential underlying or confounding mechanisms that can play a role in the bidirectional relationship between impaired carbohydrate metabolism and CLBP. Exposure to increased blood glucose levels may contribute to the occurrence, maintenance, and prognosis of CLBP and be associated with pain sensitivity via various mechanisms, including the direct impacts of glucose on the sensitivity of nociceptors [7,41], increased low-grade systemic inflammation, in particular increased tumor necrosis factor-alpha levels subsequent to raised levels of advanced glycation end products (AGEs) and oxidative stress [7,41], and atherosclerotic changes in the arteries of the spine leading to decreased blood supply [8]. An elevated PPGR is one of the main factors in glucose toxicity. Raised AGE levels subsequent to dysregulated glucose homeostasis can induce pathologic changes at the cellular and tissue level. AGEs can cause these changes via increasing oxidative stress, intervening in the functions of intracellular proteins, affecting the gene expression of certain proteins, and disrupting extracellular interactions between the matrix and cells [42]. Even a modest elevation in glucose response can increase oxidative stress biomarkers, namely ROS, at the cellular level [42]. The oxidative stress response is an internal component that has the ability to trigger inflammatory responses in the peripheral and central nervous systems, activating Toll-like receptors and changing how pain is processed [43]. Additionally, persistent exposure to increased serum glucose levels and impaired fat metabolism can have degenerative impacts on the vertebrae, cartilage, and intervertebral disks [44,45]. On the other hand, chronic (low back) pain can have a negative effect on lifestyle factors such as physical activity and nutrition, which can induce impaired glucose regulation in the body and may lead to DM2 [46].

### 4.1. Limitations and Strengths

The first limitation of this study is that PPGR was measured using a self-monitoring device using finger pricks by the researcher. Although monitoring capillary blood glucose levels with a self-monitoring device is still the most common method of analyzing blood glucose levels, it does not provide the required information to capture blood fluctuations in real-time settings. Second, as we only included women in the study, the findings of this study may only apply to women. Another limitation of this study is the absence of comprehensive data on ethnicity. While we gathered information on nationality and language demographics, specific details about race and ethnicity were absent. This deficiency could hinder the ability to apply our findings to a broader population because the diverse cultural backgrounds of participants may introduce confounding variables. Lastly, this study solely investigates the effects of sucrose and isomaltulose consumption on CLBP patients, neglecting to explore potential associations with other forms of sugar commonly consumed by the population.

The first strength of this study is the use of oral sucrose and isomaltulose load, which is a method that enables investigating the effect of GI on PPGR in isolation from confounding factors such as content of the diet, cooking methods, timing of the meal, etc. Second, both the CLBP group and HC group were similar in terms of their body composition and age, which may have had a great impact on both pain-generating mechanisms and carbohydrate metabolism. Third, the randomized cross-over design ensured high internal validity.

### 4.2. Practical Implications and Future Directions

This study’s findings have significant implications mainly for but not limited to healthcare practitioners working with CLBP patients. Elevated PPGR in normoglycemic CLBP individuals underscores the importance of monitoring PPGR during clinical assessments, given the heightened risk of impaired glucose tolerance and DM2 in this group. Implementing simple PPGR monitoring methods could aid in identifying at-risk individuals and customizing interventions. Moreover, the impact of high-GI carbohydrate intake on PPGR and its potential association with pain sensitivity suggests a need for the investigation and development of personalized dietary recommendations, emphasizing lower GI carbohydrates to manage postprandial blood glucose levels and contribute to the pain management process.

Future studies should examine the underlying mechanisms of the impaired carbohydrate metabolism and its association with pain sensitivity in patients with CLBP and explore nutritional strategies that target the quality and quantity of carbohydrates in the diet which might have a strategic and promising impact on the pain management process. Furthermore, investigations should explore this hypothesis in patients with diabetes, comparing them to healthy controls and controlling for glycemic control as indicated by glycated hemoglobin (HbA1C) levels. In addition to the content and glycemic features of certain foods, some other factors also play a role in interindividual PPGR differences such as ethnicity, gut microbiota diversity, metabolic fitness, genetics, and epigenetic markers [47]. Thus, future studies should also take into consideration these factors to ensure an individualized prediction, diagnosis, and management of impaired glucose regulation.

## 5. Conclusions

Compared to HCs, CLBP patients show higher PPGRs when consuming a high-GI beverage, namely sucrose. This finding is absent when sucrose is replaced with a low-GI beverage, isomaltulose. Thus, normoglycemic CLBP patients may have a higher risk of developing impaired glucose tolerance compared to pain-free normoglycemic individuals and might benefit more when high-GI carbohydrates are replaced with low-GI ones. In addition, we observed a positive association between self-reported pain sensitivity and PPGR to sucrose, while there was no association between any experimental pain and glycaemic response measures. Future work should examine the underlying mechanisms of impaired carbohydrate metabolism and its association with pain sensitivity in patients with CLBP and explore nutritional strategies that target the quality and quantity of carbohydrates in the diet which might have a strategic and promising impact on the pain management process.

## Figures and Tables

**Table 1:** Baseline characteristics.

	Pain Group (*n* = 53) Mean (SD)	Healthy Group (*n* = 53) Mean (SD)	Effect Size (Cohen’s d)	*p*-Value
Age (years)	37.4 (12.8)	34.1 (9.8)	0.292	0.136
Weight (kg)	72.3 (15.8)	69.7 (12.1)	0.187	0.338
Height (cm)	163.5 (6.0)	164.6 (7.1)	−0.153	0.433
BMI (kg/m^2^)	27.1 (6.1)	25.8 (4.9)	0.225	0.249
Body fat mass%	32.7 (7.1)	31.8 (6.8)	0.140	0.472
Body muscle mass %	64.0 (6.6)	64.6 (6.3)	−0.094	0.630
Body water mass %	48.2 (5.2)	48.8 (4.9)	−0.120	0.537
SF-36—PF	70.8 (20.7)	89.4 (13.0)	−1.076	<0.001 *
SF-36—RF	56.6 (40.5)	86.3 (26.2)	−0.872	<0.001 *
SF-36—SF	70.0 (25.9)	77.2 (21.9)	−0.303	0.122
SF-36—EH	57.2 (43.1)	66.6 (39.3)	−0.228	0.243
SF-36—BP	51.0 (23.9)	78.4 (20.8)	−1.224	<0.001 *
SF-36—MH	61.6 (20.4)	69.7 (15.6)	−0.445	0.024 *
SF-36—V	48.8 (20.4)	59.1 (17.7)	−0.538	0.007 *
SF-36—GH	52.7 (21.4)	65.8 (15.6)	−0.700	<0.001 *
IPAQ Low (min/week)	2747.4 (6000.1)	1620.7 (2754.3)	0.241	0.217
IPAQ Moderate (min/week)	2416.8 (2674.9)	1315.0 (1060.3)	0.542	0.006 *
IPAQ Vigorous (min/week)	1071.7 (1769.8)	588.7 (949.9)	0.340	0.083
IPAQ Total (min/week)	4643.8 (3894.4)	3589.6 (3659.3)	0.279	0.154
Mean Fasting Blood Glucose Level (mg/dL)	95.9 (8.4)	96.8 (7.8)	−0.104	0.594
Mean 2 h Blood Glucose Level (mg/dL)	96.7 (11.0)	98.3 (9.7)	−0.160	0.411
EDT	3.3 (0.6)	3.2 (0.5)	0.200	0.305
EPT	9.9 (3.9)	8.9 (3.2)	0.281	0.151
TS	22.5 (17.5)	23.0 (17.7)	−0.029	0.882
OA	6.0 (15.0)	11.4 (15.4)	−0.354	0.072
PPT—LBP	6.6 (2.1)	7.8 (3.1)	−0.470	0.017 *
PPT—TA	7.0 (2.0)	7.5 (2.7)	−0.223	0.254
CSI	42.0 (14.2)	27.2 (12.4)	1.110	<0.001 *
BPI—Severity	3.3 (1.9)	0 (0)	2.422	<0.001 *
-Worst pain during the last 24 h	4.7 (2.6)	0 (0)	2.510	<0.001 *
-Least pain during the last 24 h	2.0 (1.8)	0 (0)	1.536	<0.001 *
-Pain now	2.8 (2.4)	0 (0)	1.608	<0.001 *
-Average pain during the last 24 h	3.5 (2.0)	0 (0)	2.537	<0.001 *
BPI—Interference	3.1 (2.4)	0 (0)	1.839	<0.001 *
-General activity	3.8 (3.6)	0 (0)	1.692	<0.001 *
-Mood	3.9 (3.2)	0 (0)	1.723	<0.001 *
-Walking ability	2.8 (2.8)	0 (0)	1.409	<0.001 *
-Normal work	2.3 (2.6)	0 (0)	1.255	<0.001 *
-Relationships with others	2.3 (2.6)	0 (0)	1.253	<0.001 *
-Sleep	3.3 (3.1)	0 (0)	1.518	<0.001 *
-Enjoyment of life	3.4 (3.4)	0 (0)	1.427	<0.001 *

* *p* < 0.05, independent-sample *t*-test. *n*, number of participants; SD, standard deviation; BMI, body mass index; kg, kilograms; cm, centimeters; mg, milligrams; dL, deciliters; SF-36, short form 36; PF, physical function; RF, role function; SF, social function; EH, emotional health; BP, bodily pain; MH, mental health; V, vitality; GH, general health; IPAQ, International Physical Activity Questionnaire, min; minutes; EDT, electrical detection threshold; EPT, electrical pain threshold; TS, temporal summation; OA, offset analgesia; PPT, pressure pain threshold; LBP, low back pain; TA, tibialis anterior; CSI, central sensitization inventory; BPI, brief pain inventory.

**Table 2:** Within-group differences in IAUC.

	Sucrose Mean (SD)	Isomaltulose Mean (SD)	Effect Size (Cohen’s d)	*p*-Value
Chronic Low Back Pain (min × mg/dL) (*n* = 53)	3470.4 (1524.7)	2049.3 (942.0)	0.959	<0.001 *
Healthy Controls (min × mg/dL) (*n* = 53)	2854.9 (1147.9)	2011.1 (864.3)	0.628	<0.001 *

* *p* < 0.05, paired-sample *t*-test. *n*, number of participants; SD, standard deviation; mg, milligrams; dL, deciliters; min, minutes.

**Table 3:** Between-group differences in IAUC and difference in the amount of change when sucrose was replaced with isomaltulose.

	Pain Group (*n* = 53) Mean (SD)	Healthy Group (*n* = 53) Mean (SD)	Effect Size (Cohen’s d)	*p*-Value
Sucrose (min × mg/dL)	3470.4 (1524.7)	2854.9 (1147.9)	0.456	0.021 *
Isomaltulose (min × mg/dL)	2049.3 (942.0)	2011.1 (864.3)	0.420	0.828
Difference (min × mg/dL)	1379.7(1374.7)	844.3 (1344.3)	0.394	0.045 *

* *p* < 0.05, independent-sample *t*-test. *n*, number of participants; SD, standard deviation; mg, milligrams; dL, deciliters; min, minutes.

**Table 4:** Correlations between IAUC and pain outcome measures.

	Sucrose (*n* = 53)	Isomaltulose (*n* = 53)	Difference (*n* = 53)
EDT	−0.016	0.042	−0.022
EPT	0.070	−0.021	0.063
TS	0.151	−0.088	0.194
OA	−0.188	−0.013	−0.208
PPT—LB	−0.034	−0.075	−0.007
PPT—TA	−0.134	−0.241	−0.006
CSI	−0.171	−0.142	0.124
BPI—Severity	0.023	0.003	0.033
-Worst pain during the last 24 h	0.188	−0.99	0.238
-Least pain during the last 24 h	**0.300**	−0.114	**0.378**
-Pain now	0.234	−0.108	0.281
-Average Pain during the last 24 h	**0.394**	−0.043	**0.413**
BPI—Interference	0.147	−0.169	0.059
-General activity	**0.296**	0.110	0.260
-Mood	**0.403**	0.118	**0.347**
-Walking ability	0.134	−0.039	0.185
-Normal work	0.117	0.188	0.015
-Relationships with others	0.257	0.065	0.244
-Sleep	**0.320**	0.089	**0.296**
-Enjoyment of Life	0.269	0.048	0.269

Values shown in the matrix are Pearson correlation coefficients (two-tailed). Statistically significant values are shown in bold. *n*, number of participants; EDT, electrical detection threshold; EPT, electrical pain threshold; TS, temporal summation; OA, offset analgesia; PPT, pressure pain threshold; LBP, low back; TA, tibialis anterior; CSI, central sensitization inventory; BPI, brief pain inventory.

## Data Availability

The data that support the findings of this study are available upon request from the corresponding author, Ö.E.

## Appendix B. Description of the Elements of the Python Code

**Line Number**	**Code Element**	**Description**
2	Import module	“Import module” imports pandas and matplotlib libraries for required functions
14	for loop	Iterates through the list containing all participants’ data
17	def areaUnderCurvelbpsu	Creates a function that calculates the area under curve using the trapezoidal rule, ignoring the values below the fasting blood glucose level
19	for loop	Iterates through the list containing individual participant data
20	If statement	If all the values are higher than the fasting blood glucose level, use this function to calculate the area of the trapezoid
22	elif statement	If the first value is lower than the fasting blood glucose level and the second value is higher and equal to fasting blood glucose level, use this function to calculate the area of the trapezoid
24	elif statement	If the first value is higher than or equal to the fasting blood glucose level and the second value is lower than the fasting blood glucose level, use this function to calculate the area of the trapezoid
26	elif statement	If the fasting blood glucose level is higher than all the value, use this function to calculate the area of the trapezoid
28	sum variable	Total sum of the area under the curve value for each individual
